# The work environment pilot: An experiment to determine the optimal office design for a technology company

**DOI:** 10.1371/journal.pone.0232943

**Published:** 2020-05-19

**Authors:** Jegar Pitchforth, Elizabeth Nelson-White, Marc van den Helder, Wouter Oosting

**Affiliations:** 1 Booking.com, Amsterdam, Netherlands; 2 LearnAdaptBuild, Amsterdam, Netherlands; 3 CBRE Consulting, Amsterdam, Netherlands; Jonkoping University, SWEDEN

## Abstract

Over the past few decades many corporate organisations have moved to open-plan office designs, mostly due to financial and logistical benefits. However, recent studies have found significant drawbacks to open plan offices and it is unclear how office designs can facilitate the best work output and company culture. Current design practice aims to optimise efficiency of space, but no previous research has tested the effect of office design experimentally in a working office. This paper describes an experiment comparing four different office designs (Open-plan, Zoned open-plan, Activity based, and Team offices) against a suite of wellbeing and productivity metrics in a real world technology company. Results suggest that two very different designs (Zoned open-plan and Team offices) perform well compared to Open-plan office designs. Zoned open-plan and Team office designs improved employee satisfaction, enjoyment, flow, and productivity, while Activity based and Open-plan designs performed poorly by comparison. The Open-plan office design was rated more poorly by employees, had higher levels of unsafe noise, and once employees no longer had to be in the Open-plan office design of the experiment, they spent more time at their desks.

## Introduction

Office design is an important factor in many variables of interest to managers, but the impact of open office design has rarely been explored experimentally. Since open offices have become mainstream, research has questioned their efficiency and general likability. Recent research has even gone so far as to wholly condemn open office designs, but designers are working with creative ideas to improve the open workplace [1]. To date, there has been no randomized, controlled trial in a working organisation capable of establishing such causal relationships. This paper presents the first randomized, controlled experiment in a working international technology company with the aim of finding an optimal open office design for employees.

### Office design challenges

Office design can be a source of satisfaction [2, 3], engagement [4, 5], productivity [6, 7], and employee health [8, 9], but how companies design an office to meet the needs of their organisation is an open question. In the past, this question has frequently been answered through corporate strategy and budget restrictions [10]. However, as organisations become more data-driven and office space costs increase, many companies are beginning to consider other types of costs such as loss of productivity and decreased employee engagement [11]. These considerations have led to new questions regarding the impact of office design choices on employees.

A number of different office designs have been used over time to facilitate a wide range of work styles and goals. In this study we examine four designs common to modern companies, here described as *Activity based*, *Open-plan* (our control design), *Zoned open-plan* and *Team office*.

Activity based offices are flexible zone-based environments with unassigned seating that provide a range of spaces intended for different usages in an open setting. They have become popular due to aspects such as space reduction and cost savings, with employees making more efficient use of the office floorspace. Despite the financial benefits of the approach, activity based designs have been shown to decrease comfort, privacy and productivity [12] and contribute to emotional exhaustion [13]. Further, satisfaction with activity based environments is limited to one subset of workers [14]. However, recent work [15] has suggested that some of these issues can be overcome by including employees in the process of changing from their existing environment to an activity based environment. These mixed findings suggest that there is more that can be learned about this office design approach through experimental observation.

Open-plan offices are designed with minimal separation of spaces, such that the office floor is without internal walls or doors. They are currently very popular in large corporations [11], but they are associated with a range of issues including increased disturbances and lack of privacy [16–19]. Past research has highlighted the tendency for open-plan office designs to drive negative behaviors and attitudes of employees through loss of space and increased contact with coworkers [17, 19–23]. It has been established that environmental variables such as noise and visual disturbances [24, 25], poor air quality [26], temperature [27], and lighting [28] have an impact on satisfaction, engagement, and productivity in open plan environments, suggesting that examining open-plan office design in an experimental context adds value to the literature.

In contrast to Open-plan offices, cubicles are a way of breaking up open office floors with partitions between desks, providing an enclosed desk space for each employee. The design was once the most common type of office design, but has become less popular in recent years [29]. Some benefits of this type of office design, such as reduced visual distraction leading to increased perseverance [30] have been identified, but these are now seen as being offset by negative cultural effects and reduced collaboration [31, 32]. Most work on this design has focussed on individual cubicles, with no known work on designs with a team of employees in a single large cubicle.

While many researchers have chosen to frame their work in terms of different office design styles, others have remained more general in their approach and have instead focussed on relationships between specific environmental and commercially important variables. Here we review some variables that are proposed to be affected by office design.

### Environmental variables

Sound disturbances have been documented to reduce cognitive performance, decrease motivation, and increase stress levels [24, 33–36]. The prevalence of both audio and visual disturbances have flagged the need for improving employee privacy in the workplace [19].

Other environmental aspects of workplaces such as temperature, air quality and light have shown both negative mental and physical reactions to sub-optimal environments [37, 38]. The term ‘sick building syndrome’ was coined over 30 years ago when it was discovered that many of the offices we work in had poor air quality and were making people ill [37]. The 1984 World Health Organization (WHO) report suggested that up to 30% of new and remodeled buildings worldwide may be subject to complaints related to poor indoor air quality [39].

Office temperature has been established to be linked to both productivity [27, 40] and satisfaction [41, 42]. Recent studies have found that around 22 degrees appears to be an optimal temperature [27, 43, 44]. While there are many academic, government, and industry organisations specifying optimal office temperatures [45–47], there is still debate around the specifics of how temperature affects employees. For example, [48] found that the relationship between temperature and performance was different for different types of work, and [49] found that women perform better on some types of work in higher temperatures than men. It may be that there is so much variation in reactions to office temperature that finding one optimal temperature is not possible [50]. However, given the ability to control office temperature and relative ease of measurement, it is of great value to understand more about how this variable affects employees.

Lighting is another important aspect of office design that can have significant effects on employees. The effects of different intensity, colour, and positioning of lighting on employees have been established in a number of studies [18, 51, 52], although there at present is no conclusive method of designing an optimal office lighting plan. Much of the previous work has focussed on how light can affect productivity by affecting circadian rhythms and making employees drowsy [52], and results suggest lighting states similar to natural light at the end of the day should be avoided. Recently [53] proposed a more technologically based solution involving estimating employee drowsiness, then adjusting lighting and air conditioning accordingly. With such a strong link established but little work on experimental observation outside a laboratory environment, there is still much to be learned about the effect of this variable on employee behaviour.

### Occupancy and personal variables

Occupancy refers to the number of people using a space, and is an important factor in office operations planning [54, 55] as it affects a wide range of decisions such as required cleaning staff and opening hours. In academic literature, the primary focus of collecting occupancy data through sensors has been for further modelling purposes [56, 57] but has not been examined in relation to environmental variables such as temperature, noise, or air quality.

Productivity is the primary variable of interest to most organisations, as it is the source of profit and innovation. Given that productivity can mean different things in different job roles, it is easy to see why most studies of productivity have focussed on heavily operational roles with clear productivity metrics such as sales [58, 59], manufacturing [60, 61], or nursing [62–64]. A notable exception is the work of [65], who examined the relationship between work habits and productivity in software developers. They note that software developer productivity is very difficult to measure, and that in such a situation it is more useful to measure perceived productivity. While this method may be less accurate than observing metrics of productivity, it is likely to be more relevant for modern knowledge industry companies. A measure for perceived productivity based on role and identity theory was introduced by [66], and has since been used in a variety of work areas such as public service [67] and nursing [68].

One tool commonly proposed as a proxy for productivity in technology driven companies is Git, a software first introduced in 2012 [69] which assists developers in coding productivity at a group level. The framework allows developers to ‘commit’ their code to collectively managed code bases in a systematic and well-recorded fashion. Scholtes in 2016 [70], demonstrated that logs of git activity can usefully be examined in relation to productivity. [71] raised the concern that git activity could be misleading given the software’s potential weakness to bad actors and issues with transporting repositories. These issues are not a concern in the current study however, as the corporate environment serves as a protection from both bad actors and infrastructure problems.

Satisfaction is another important concept for organisational and office design research, and is used as a primary measurement for the sentiment of employees. A common measurement of satisfaction with the working environment is the Leesman satisfaction index [72], which is seen as useful for benchmarking purposes. While satisfaction is clearly related to organisational and operational factors such as management styles [73–75] and job requirements [76, 77], there is a large body of work that explores satisfaction with reference to office design. [78] found that satisfaction was related to view of nature, and [14] suggested that offices requiring more place-switching provided a higher level of satisfaction. In addition to the field-studies on satisfaction in office designs [79, 80], there have been a number of quasi-experiments examining employee satisfaction before and after office design changes [15, 80]. These studies show that satisfaction is of high interest to both researchers and corporations, but the construct is yet to be explored experimentally in the context of working technology companies.

Since its introduction in the literature, managers have been interested in how to help their employees reach psychological flow states, as these are seen to be related to productivity [81, 82]. The concept of flow stems from the positive psychology literature [83, 84], which uses the term to describe an optimal mental state for accomplishing a task. [85] outlines the various methods for measuring flow in different environments, such as questionnaire-based scales such as the WOLF scale [86], or the Experience Sampling Methodology [87] which requires frequent sampling at an individual level [88]. Hypothesised drivers of flow include task challenge [89], daily recovery [90], job characteristics [91], and organisational and personal resources [92], but no solid theory has yet been established on this topic. There is very little work on the relationship between office design and flow states, with recent work by [93] a notable exception, who invoke flow as one of the benefits of their proposed Emotional Design approach. [90] explored both energy at work and flow, focusing on the role of ‘detaching’ from work tasks outside of working hours.

Engagement (or its opposite, burnout), and enjoyment are closely related concepts that are also proposed to be affected by office design. Burnout is often described as the antipode of engagement [94], and has been noted to cause significant costs to businesses in the form of lost productivity [95]. Recently, it has become more convenient to measure burnout with the introduction of Maslach’s Burnout Inventory [96] a tool which is used to diagnose burnout in countries like The Netherlands, where this study was conducted. While previous research has focussed largely on effects of more abstract burnout drivers such as job role [97, 98] or social environment [99, 100], relatively little attention has been paid to the effects of the physical work environment on burnout [101, 102].

Enjoyment of a space is also very useful for organisations to measure, as it is related to other staff behaviours such as productivity and wellbeing [103]. Enjoyable office spaces can improve activity of sedentary workers [104], help guide corporate culture [105], and improve creativity [106]. While studies have demonstrated a clear relationship between enjoyment of office spaces and benefits to organisations, there is still a need to examine how different office designs vary in enjoyment from an experimental paradigm.

Studies of open office designs have considered these variables observationally, but there is still a notable gap in the evidence from systematic experimentation in real corporate environments. Previous research has explored aspects of employee sentiment and behaviour in relation to various aspects of the work environment, but none have employed an experimental design, tending to prefer quasi-experimental (non-randomized) and case study approaches such as work by Cisco [107] and Hewlett-Packard [108]. The present paper describes the first experimental analysis of these four open office designs in direct comparison. It also takes a multivariate approach to understanding employee behaviour, with analysis based on automated data collection in addition to more commonly used research tools such as questionnaires and interviews. By systematically observing the performance of four common open office designs in a modern technology focussed company, we demonstrate that an experimental approach to office design can help corporations find optimal office designs and design elements for their employee population, as well as providing evidence of the relationships between office design and commonly studied constructs with strong ecological validity.

Given the current state of office design research and the noted gap in the literature, the aim of this research is to use an experimental approach to compare four open office designs, and determine if there is an optimal open office design which fosters productive work but also reaches high levels of satisfaction for employees of a large corporate tech company.

## Materials and methods

This study involved human subjects, and was formally approved with written confirmation by the Booking.com B.V. Works Council in the Netherlands, which is a legally designated body charged with upholding ethical and professional standards for the company. All data were analyzed anonymously in accordance with European GDPR regulations.

Booking.com is a large corporate tech company founded 23 years ago in the Netherlands with an employee population of 17,500 people globally, 5,580 of which are based in the global headquarters in Amsterdam. Their employee population includes software and system developers, travel market specialists, marketing, finance, and a range of support roles facilitating the entire customer experience.

### Participants

Participants were recruited as whole teams from a sample of all departments and job roles in Booking.com, excluding Customer Service agents who work in call centers rather than an office environment. The final sample included 288 participants from 22 teams in a similar mix of functions to that found in the wider company including code development, project management, administration of HR processes, and strategy formulation. Each team ranged in size from four members to 20 members.

All participants were informed of the intent to run an experiment and were shown outlines of the environment they would be seated in. They discussed their participation in the experiment with their team, who decided to participate or not. Communications to participants stressed that there was no requirement to participate in the experiment, and that there would be no consequences for non-participation. The experimental area was clearly marked with signage indicating the boundaries of the experiment, and that participants could leave the area if they did not want to participate. All experiment plans were presented to the Booking.com Works Council, a legally mandated body with oversight of all changes regarding employees, who provided written consent on behalf of the employee population.

**Fig 1:**
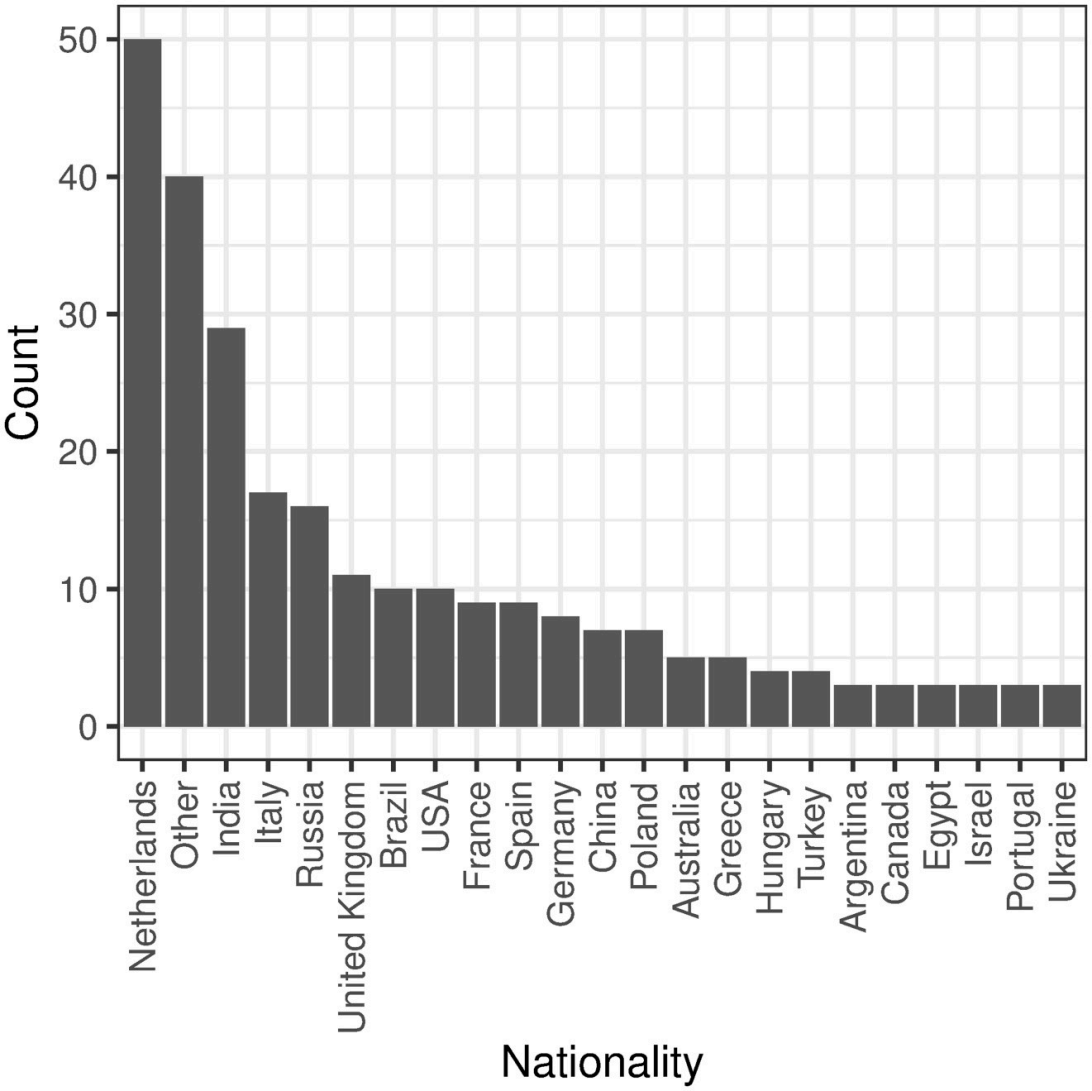
Nationality of participants.

Participants in the sample ranged in age from 22–59 years old with a mean age of 32. The gender split loosely reflected the company population with 59.84% identifying as Male. 51 nationalities were represented, including Asia, Europe, North and South America, Africa, and Oceania (Fig 1).

### Sample selection

Sampling was achieved using a stratified convenience sampling approach wherein teams were asked to volunteer for the experiment, with a preference for complete organisational sets of teams (known as ‘tracks’). Teams were selected from those that volunteered to participate, working closely with team and track managers to ensure full coverage of job roles. The final choice of participants ensured representation was proportional to the makeup of departments in the company.

### Experiment design

A uniform cross-over design [109–112] was employed to systematically observe the effect of office design on the constructs of interest. The specifics of the design and consequent analysis method is described below.

#### Blocking and randomized exposure

Teams of participants were assigned to one of four groups. An R script was used to randomly assign teams to groups such that each group had equal numbers of individual participants.

Each group was then exposed to each office design for two weeks at a time before moving to the next design. The sequence of exposure was randomized such that no group was exposed to the designs in the same order as any other group. See Table 1 for the exposure schedule adopted.

**Table 1:** Latin Square [110] exposure schedule starting with random assignment of groups to designs, then arranging assignment such that each group was exposed to each design in a different order. Letters A through D represent the group assigned to that design for that wave.

	Design			
Wave	Open plan	Zoned open plan	Activity based	Team offices
1	A	C	D	B
2	C	B	A	D
3	D	A	B	C
4	B	D	C	A

Scheduling the exposure pattern for groups in this way ensured that sequence effects and time effects were both controlled for when the experiment was analysed over all four waves. This assumption is explicitly validated in the analysis phase.

#### Office designs

Office designs were designed in workshops with a working group of 10 employees led by Booking.com’s Real Estate team and CBRE, a commercial workplace design consultancy. The overall aim of the process was stated to be ‘to design an office space that is healthier, happier, and more productive’. Using a heuristic needs gathering process, three office designs were developed for testing that were proposed to suit the working style of the employee population. With the addition of the Open-plan office design as a control design, four designs were chosen for testing in the final experiment.

**Fig 2:**
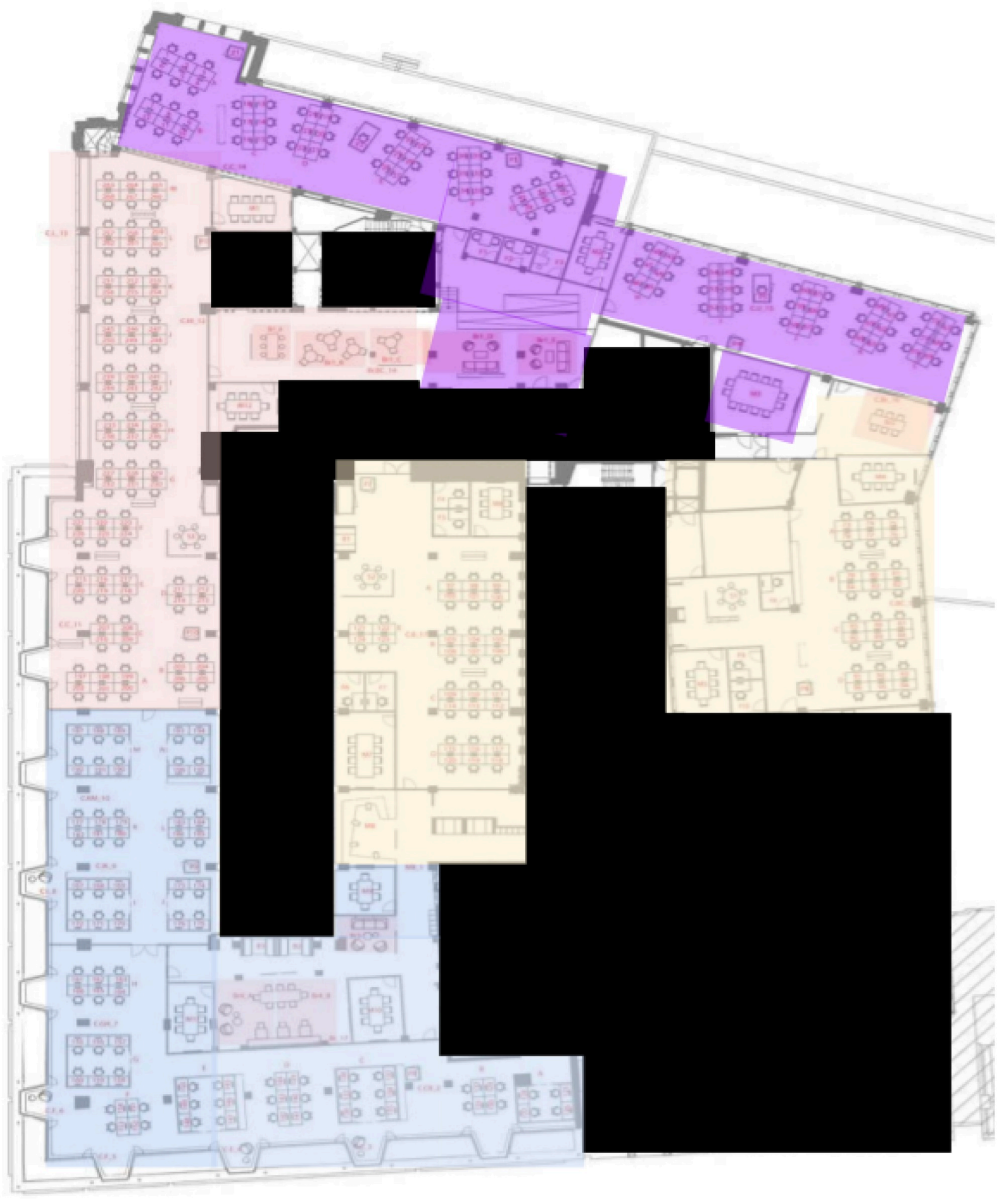
Floorplan of the experimental floor. Blacked out areas represent empty spaces (e.g. atriums) and non-experimental areas. Coloured areas represent experimental zones Control (red), Limited open plan (purple), Zoned open plan (yellow), and Team offices (blue).

All office designs were implemented on the same floor of a single building in Amsterdam such that approximately a quarter of the available floor space was occupied by each design (see Fig 2 for a floor plan of the experimental area). Participants were informed through a variety of communication channels that this area was being monitored through sensors, and that they were able to opt out by leaving the space.

**Fig 3:**
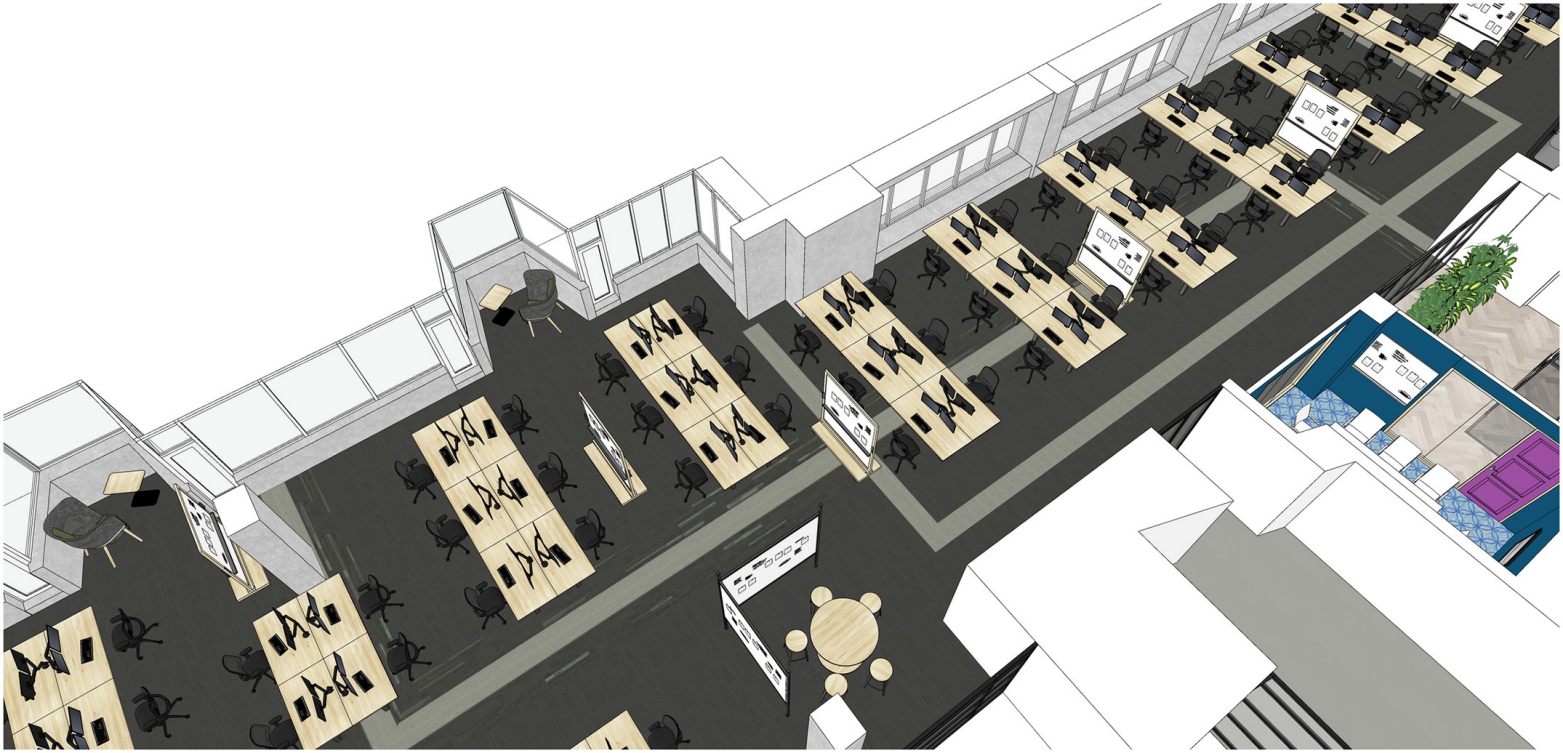
Open-plan (Control). Digital render of the Open-plan Control area.

**Fig 4:**
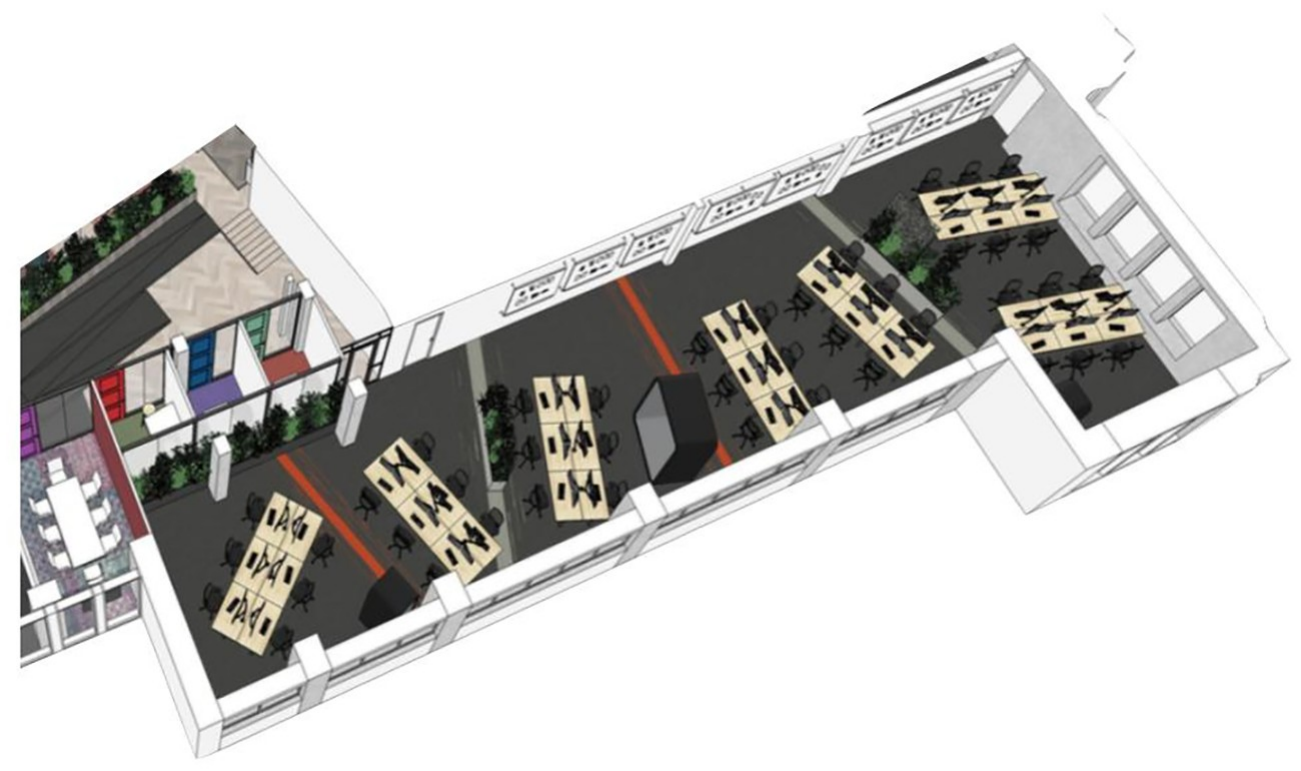
Zoned open plan. Digital render of the Zoned Open-plan design.

**Fig 5:**
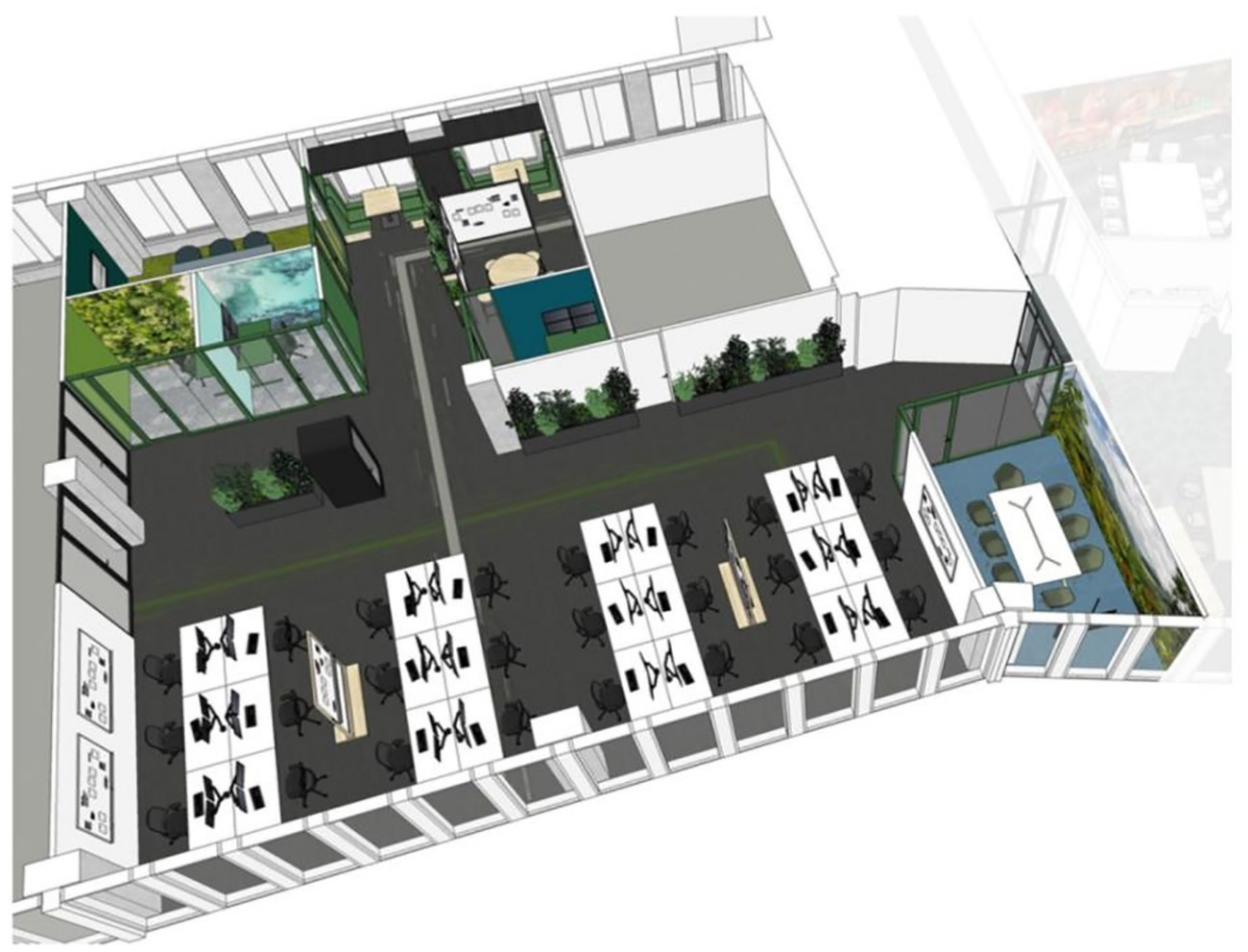
Activity-based design. Digital render of the Activity-based design.

**Fig 6:**
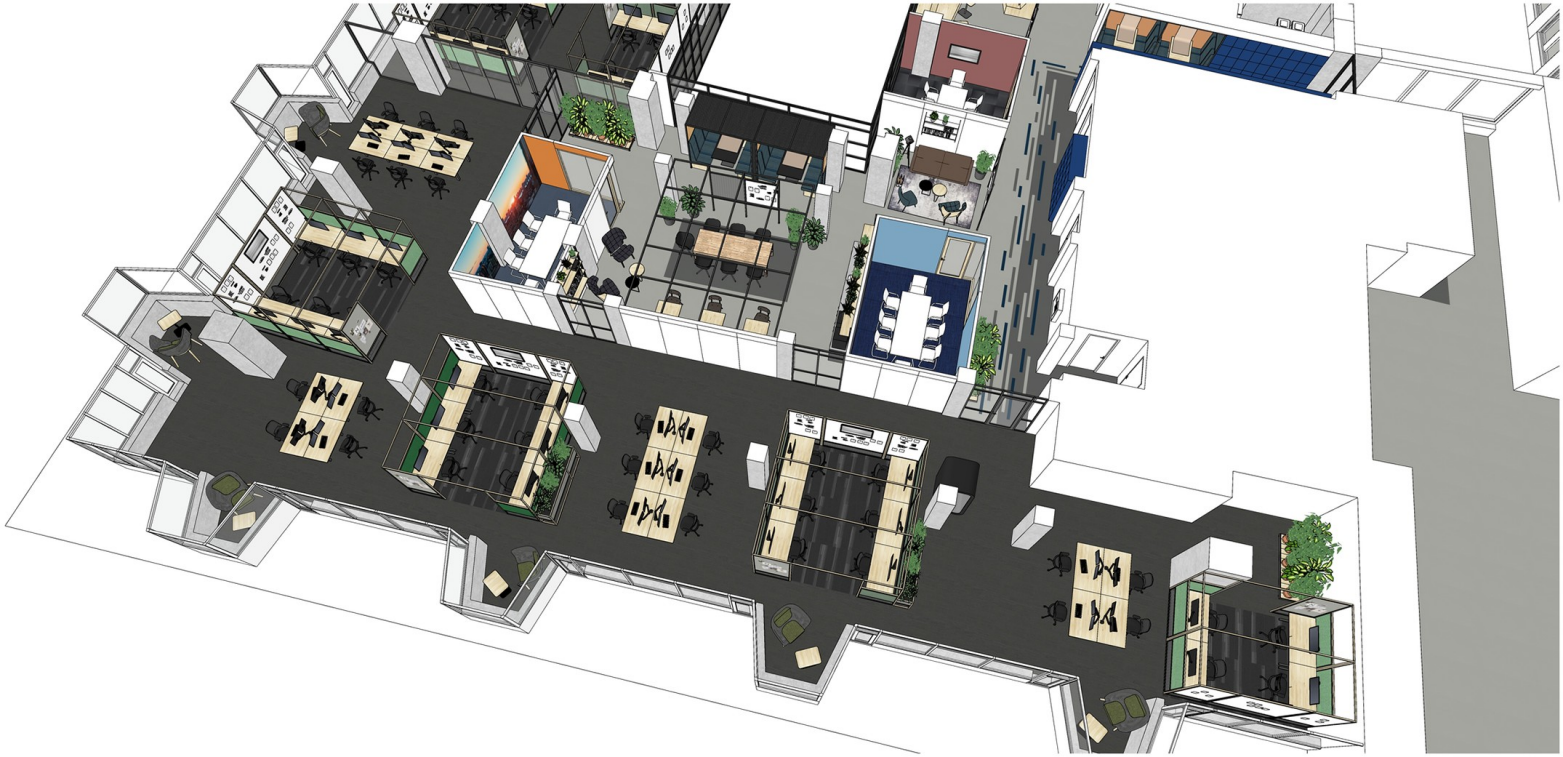
Team office design. Digital render of the Team office design.

1. Open-plan (Control)
  The Open-plan design is very similar to many large technology based companies around the world, and serves as a control design for this study (Fig 3). In the present study it is an open-plan design with groups of six desks, allowing three people to sit on either side.
2. Zoned open-plan
  The Zoned open-plan design was proposed initially by project architects as their first answer to the needs gathering process. Zoned open-plan is similar to the open-plan design for working spaces, but added soundproof doors between working and collaboration spaces (Fig 4). Each Zoned open-plan zone had no more than 40 people in a room (compared to a maximum of 72 occupants), and each room included at least two ‘phone booth’ style units with soundproof doors. Plants were integrated into the space using hanging planter boxes above each set of desks.
3. Activity based
  The Activity based design is an open-plan design in which desks are not officially assigned to a specific employee and includes activity-centered zones (Fig 5). Other spaces are provided such as small, one person rooms with desks and screens (known as ‘focus rooms’), phone booths, and a variety of collaboration spaces of different sizes and levels of privacy.
4. Team office
  The Team office design is the closest to the traditional design of the cubicle, with each cubicle large enough to sit six or four people(Fig 6). Team offices each contained six of four desks, and included a whiteboard and large screen for sharing content. Each space was delineated by walls of sound absorbing panels.

### Constructs

The experiment measured outcomes over 9 constructs. Operational definitions are defined in Table 2.

**Table 2:** Definitions and measurements of constructs considered in the experiment.

Construct	Operational Definition	Measurement
Satisfaction	The extent to which the design fulfilled the needs and wishes of employees	Survey items
Engagement	The extent to which participants felt focussed on and excited to complete regular work tasks.	Survey items
Enjoyment	The extent to which participants took pleasure in being in the design space.	Survey items
Environment	The proportion of time which each environmental variable was within a healthy range.	Sensor data
Energy	The level of motivation experienced by the participant.	Survey items
Flow	The extent to which the participant associated with descriptions of flow states.	Survey items
Productivity	The extent to which the participant felt they were able to complete their work.	Survey items
Commits	The number of commits submitted to the main git repository.	Git logs
Occupancy	The proportion of desks that were occupied during working hours.	Sensor data

Healthy ranges for environmental variables were chosen based on suggestions from the literature [45–47]. Table 3 contains the final ranges that were chosen for this experiment.

**Table 3:** Ranges of environmental variables considered to be healthy in this experiment.

Variable	Healthy Range
Temperature	20-24 c
Light	500-1000 lux
Noise	<70 db
Humidity	30%–70%
CO_2_	<2050 ppm

A generalized linear model is adopted for the analysis of each of the outcome measures, stating the effect of time, group membership, carryover effect, and treatment effect explicitly [109, 110]. The model is formally defined as:

$$\begin{array}{c} g\left(E\left(Y_{ij}\right)\right)=g\left(\mu_{ij}\right)=\mu +\pi_{i}+s_{j}+\tau_{d(i,j)}+\rho_{d(i-1,j)}+\beta_{1}x_{1}+\ldots +\beta_{m}x_{m} \end{array}$$ (1)

where:

*g* = The appropriate link function for the response distribution.

*Y*_*ij*_ = The value of outcome variable *Y* for time *i* in group *j*.

*μ* = The overall response mean.

*π*_*i*_ = The effect of time *i*.

*s*_*j*_ = The effect of the *j*^*th*^ group.

*τ*_*d*(*i*, *j*)_ = The effect of the design *d* at period *i* to group *j*. Design *d* corresponds to the designs referred to in Table 1.

*ρ*_*d*(*i*−1,*j*)_ = The carryover effect applied at period *i* − 1 to the group *j*, under the design *d*.

*β*_*m*_ = The effect of covariate *m* on the outcome Y.

For perception based outcomes, the covariates introduced to the model were based on demographic features that were believed to be of importance to determining the experience of the office space, such as age, gender, workgroup (i.e. company department), nationality, introversion, preference for morning or evening work, and the level of collaboration required to work.

Most response variables were normally distributed, and thus most models are linear models (identity link). Git commits were Poisson distributed, so a log link function is chosen. For each outcome the overall effect of each term is examined using an ANOVA. In cases where office design has a significant effect on the outcome variable, we examine the coefficients of the generalised linear model for all terms that were shown to have a significant effect on improving the fit of the model to data.

### Materials

#### Sensors

Two brands of sensor, ERS (82 sensors) and PointGrab CogniPoint (282 sensors), were used to measure environmental and behavioural variables. Each sensor type was administered by a third party sensor specialist who collected and collated the data before sending it for analysis by Booking.com Data Scientists. Sensor locations were recorded by the installation company as a map, then these points were manually input to the QGIS software [113] to ensure each set of readings was associated with specific coordinates.

ERS sensors [114] measure a set of environmental variables (light, temperature, noise, movement, CO_2_ levels) simultaneously, and send these measurements to a receiver which in turn transmits data to servers. This process occurs regularly at 15 minute intervals.

PointGrab CogniPoint [115] sensors are used to sense desk occupancy. These sensors contain infrared cameras and have functionality to set a zone of interest within the video frame. They produce a count of the number of people seen within the zone of interest once every 5 minutes.

#### Questionnaire

A questionnaire was administered to all participants at the end of each wave of exposure. All responses were required to be anonymous, so respondents provided the design to which they were assigned for that wave and responses were analysed at the group level. Differences between groups were controlled by the repeated-measures experiment design.

The questionnaire was designed based on past studies measuring both individual outcomes as well as group outcomes of environmental change. Satisfaction and perceived productivity were measured with 1 item, engagement with 3 items, enjoyment with 3 items, energy with 3 items, and flow with 4 items (see Table 4).

**Table 4:** Measurement items.

Construct	Item
Satisfaction	Overall how would you rate this workplace concept?
Productivity	This workplace enables us to work productively.
Engagement	At my work, I feel full of energy.
Engagement	I am enthusiastic about my job.
Engagement	I am immersed in my work.
Enjoyment	This workplace creates an enjoyable environment to work in.
Enjoyment	This workplace contributes to a sense of community at work.
Enjoyment	This workplace is a place I’m proud to bring visitors to.
Energy	I am consistently tired.
Energy	I feel emotionally drained from my work.
Energy	I have become less enthusiastic about my work.
Flow	My mind wasn’t wandering, I wasn’t thinking of something else.
Flow	I was totally involved in what I was doing.
Flow	Movement of colleagues didn’t disrupt my work.
Flow	I worked without interruption from background noise or colleagues needing to talk.

#### Git commits

Git commits were collected as a loose proxy for technical output. This was achieved using a bash script that was run once at the end of the project using:

git shortlog -sne

with some extra parameters to filter counts for only experimental participants.

### Data analysis and preparation

Data were analysed using R based on CSV files from sensor providers (for environmental measurements) and Qualtrics [116] (for survey responses). Data from each wave were engineered into a database in Hive to allow for modelling. Sensors were hung in the space for 2 weeks during a beta test to see both functionality of the devices, results of the space and behavior. It was established during this time that sensor measurements could be limited to 8 AM to 6 PM on weekdays, as no employees were in the office outside those times. This was confirmed in a check of the data during the experiment.

Occupancy data were aggregated by hour to calculate the average proportion of occupancy per design for that period. This decision was made to normalise the number of measurements taken per period, as each sensor provided readings asynchronously (such that there was a different number of readings per sensor within each hour window) and due to the Activity based design having a lower number of occupancy sensors in the design (due to the desk-sharing ratio introduced). In addition, a binomial treatment of the data would have considered each measurement to be independent, which is not the case for occupancy data taken from sensors, where each reading is both time and sensor dependent. Following an assessment of the data choices, the hourly aggregation method was considered most actionable by stakeholders. Analyses of the data in both raw and aggregated forms are provided in the supporting data for the reader’s convenience.

## Results

### Questionnaire

**Fig 7:**
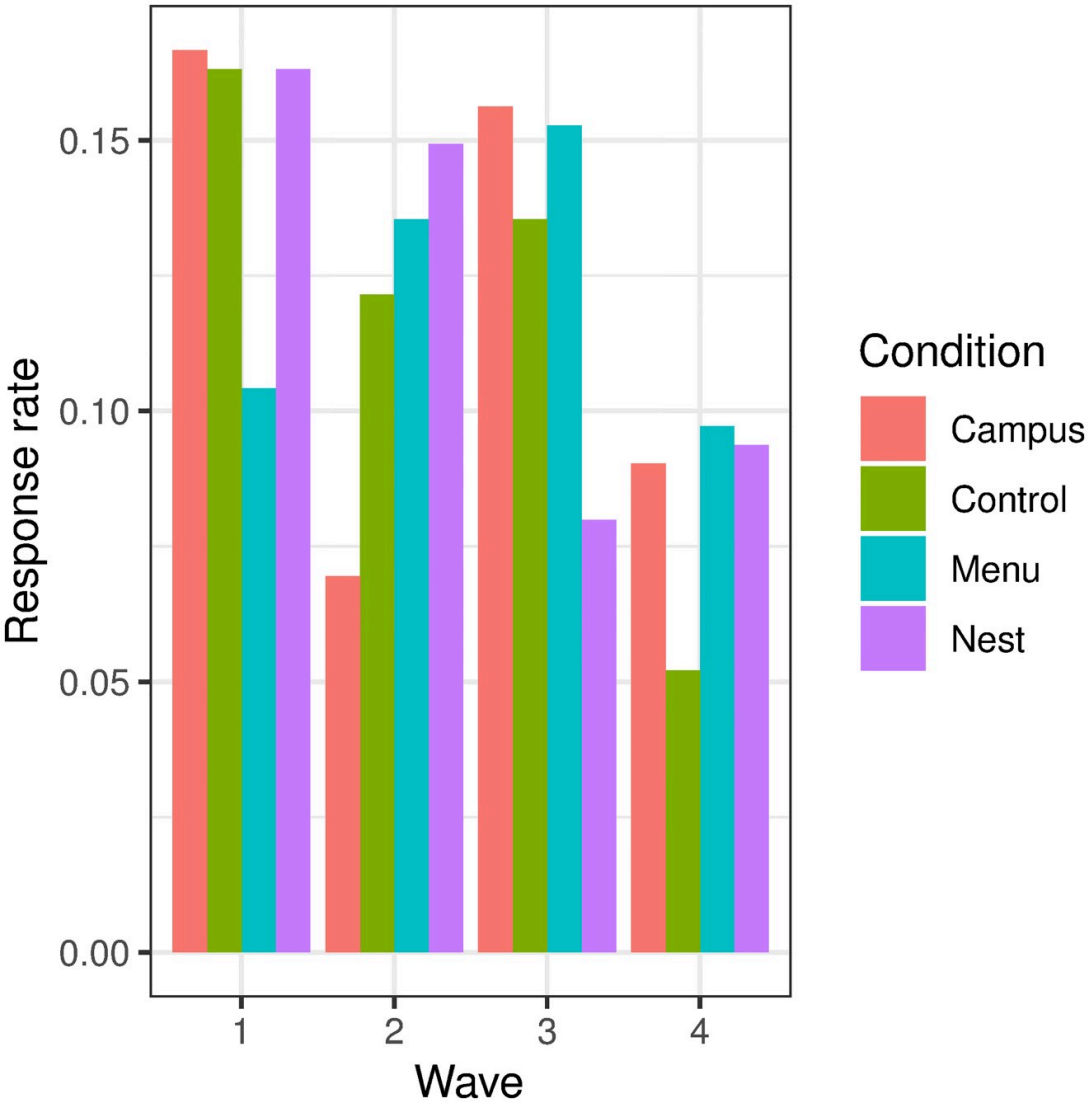
Response rates. The response rate for each survey wave by design.

The questionnaire had a declining response rate over the course of the experiment (Fig 7). As responses were to be analysed at the group level, this decrease in responses over time was not considered to be a major problem as time is controlled through the repeated measures experiment design.

For each outcome a GLM was used to determine the effect of the experimental design on that outcome while also controlling for covariates. The effect of experimental design was found to be significant for all outcomes except for energy and commits, which are subsequently excluded from reporting in results. Covariates with significant effects are reported here, and non-significant effects are reported in the supplementary information.

#### Satisfaction

A linear model revealed a significant effect of office design on participant satisfaction with the workplace (*F* = 39.958, *p* < 0.001) (Table 5). Participants reported 12% higher satisfaction in the Zoned open-plan design (*est*. = 1.169, *se* = 0.295, *p* < 0.001) and 8% higher satisfaction in the Team office design (*est*. = 0.781, *se* = 0.287, *p* = 0.007) than in the Open-plan design (Table 6). Participants reported 14% lower satisfaction in the Activity based design (*est*. = -1.391, *se* = 0.307, *p* < 0.001) than in the Open-plan design.

**Table 5:** ANOVA table for satisfaction.

	Df	Deviance	Resid. Df	Resid. Dev	F	Pr(>F)
NULL			501	2754.77		
area	3	517.81	498	2236.96	40.07	0.0000
group	3	32.80	495	2204.15	2.54	0.0560
previous	4	33.13	491	2171.03	1.92	0.1055
Gender	1	10.99	490	2160.04	2.55	0.1109
Age	1	0.00	489	2160.04	0.00	0.9885
Nationality	10	72.83	479	2087.21	1.69	0.0801
Workgroup	6	25.86	473	2061.35	1.00	0.4242
Introversion	1	0.07	472	2061.28	0.02	0.8993
Preferred time	1	16.16	471	2045.12	3.75	0.0534
Works_noise	1	0.38	470	2044.75	0.09	0.7675
Work_collagues	2	7.43	468	2037.32	0.86	0.4227
Days_concept	1	25.74	467	2011.58	5.98	0.0149

**Table 6:** GLM results for satisfaction.

	Estimate	Std. Error	t value	Pr(>|t|)
(Intercept)	5.0253	1.0882	4.62	0.0000
areaZONE	1.1894	0.2943	4.04	0.0001
areaACTIVE	-1.3701	0.3057	-4.48	0.0000
areaTEAM	0.7836	0.2873	2.73	0.0066
group2	0.5479	0.3846	1.42	0.1550
group3	0.4441	0.3333	1.33	0.1835
group4	0.1833	0.3517	0.52	0.6024
previousControl	0.3146	0.3432	0.92	0.3598
previousACTIVE	0.1232	0.3666	0.34	0.7369
previousTEAM	-0.0519	0.3750	-0.14	0.8899
previousNone	0.5976	0.3140	1.90	0.0576
Gender2	0.3357	0.2185	1.54	0.1252
Age	0.0078	0.0183	0.43	0.6708
Nationality2	-0.4343	0.6731	-0.65	0.5191
Nationality3	0.5338	0.7624	0.70	0.4842
Nationality4	-0.0939	0.6033	-0.16	0.8764
Nationality5	-0.6598	0.6506	-1.01	0.3110
Nationality6	0.2826	0.9067	0.31	0.7554
Nationality7	0.6665	1.2280	0.54	0.5876
Nationality8	-0.8260	0.6817	-1.21	0.2263
Nationality9	0.1167	0.6648	0.18	0.8607
Nationality10	-0.7757	0.8833	-0.88	0.3803
Nationality11	-0.7795	0.7401	-1.05	0.2928
Workgroup2	0.2603	0.4983	0.52	0.6017
Workgroup3	0.4933	0.3432	1.44	0.1513
Workgroup4	0.0963	0.3704	0.26	0.7951
Workgroup5	0.0511	0.3895	0.13	0.8957
Workgroup6	0.4863	0.3857	1.26	0.2081
Workgroup7	-0.3637	1.1100	-0.33	0.7433
Introversion	-0.0272	0.1578	-0.17	0.8635
Preferred time	-0.2457	0.1199	-2.05	0.0409
Works_noise	-0.0815	0.1879	-0.43	0.6646
Work_collagues2	-0.0245	0.2750	-0.09	0.9291
Work_collagues3	0.3032	0.3278	0.93	0.3554
Days_concept	0.2933	0.1200	2.44	0.0149

Of the covariates, only the number of days in the concept had a significant effect on satisfaction (*F* = 6.275, *p* = 0.012). Further exploration of the covariates revealed that each additional day participants had spent in the concept was associated with 3% higher satisfaction (*est*. = .303, *se* = 0.121, *p* = 0.012).

#### Engagement

A linear model revealed a significant effect of office design on employee engagement in the workspace (*F* = 3.279, *p* = 0.0208) (Table 7). Participants reported 7% higher engagement in the Team office design than in the Open-plan design (*est*. = 0.701, *se* = 0.293, *p* = 0.017). The Activity based and Zoned open-plan designs did not receive significantly different engagement scores to the Open-plan design (*est*. = -0.383, *se* = 0.312, *p* = 0.221, and *est*. = 0.461, *se* = 0.301, *p* = 0.126 respectively).

**Table 7:** ANOVA table for engagement.

	Df	Deviance	Resid. Df	Resid. Dev	F	Pr(>F)
NULL			507	2568.21		
area	3	44.37	504	2523.84	3.29	0.0207
group	3	35.21	501	2488.63	2.61	0.0511
previous	4	40.83	497	2447.79	2.27	0.0610
Gender	1	4.25	496	2443.54	0.95	0.3314
Age	1	33.50	495	2410.04	7.44	0.0066
Nationality	10	42.20	485	2367.84	0.94	0.4981
Workgroup	6	73.80	479	2294.03	2.73	0.0128
Introversion	1	87.74	478	2206.29	19.49	0.0000
Preferred time	1	27.80	477	2178.49	6.18	0.0133
Works_noise	1	2.22	476	2176.27	0.49	0.4827
Work_collagues	2	42.61	474	2133.67	4.73	0.0092
Days_concept	1	4.56	473	2129.11	1.01	0.3149

Of the covariates, age (*F* = 7.425, *p* = 0.007), workgroup (*F* = 2.726, *p* = 0.013), introversion (*F* = 9.763, *p* < 0.001), preferred time of day to work (*F* = 3.71, *p* < 0.001), and the level of collaboration required for participants’ tasks (*F* = 4.591, *p* = 0.011) all had significant effects on engagement (Table 8).

**Table 8:** GLM results for engagement.

	Estimate	Std. Error	t value	Pr(>|t|)
(Intercept)	4.6995	1.0941	4.30	0.0000
areaZONE	0.4704	0.2993	1.57	0.1167
areaACTIVE	-0.3821	0.3110	-1.23	0.2198
areaTEAM	0.7012	0.2924	2.40	0.0169
group2	1.0555	0.3910	2.70	0.0072
group3	0.2556	0.3390	0.75	0.4513
group4	0.2669	0.3574	0.75	0.4555
previousControl	-0.4125	0.3504	-1.18	0.2397
previousACTIVE	-0.6095	0.3738	-1.63	0.1036
previousTEAM	-0.0905	0.3814	-0.24	0.8126
previousNone	0.2055	0.3195	0.64	0.5204
Gender2	0.1208	0.2218	0.54	0.5863
Age	0.0478	0.0186	2.57	0.0105
Nationality2	-0.6120	0.6654	-0.92	0.3581
Nationality3	-0.1647	0.7516	-0.22	0.8266
Nationality4	-0.6430	0.5920	-1.09	0.2780
Nationality5	-0.6595	0.6394	-1.03	0.3029
Nationality6	-0.5958	0.9135	-0.65	0.5146
Nationality7	-0.3205	1.2431	-0.26	0.7967
Nationality8	-0.8978	0.6736	-1.33	0.1832
Nationality9	-0.3894	0.6559	-0.59	0.5529
Nationality10	-1.2779	0.8871	-1.44	0.1504
Nationality11	0.1714	0.7358	0.23	0.8159
Workgroup2	-0.3511	0.5029	-0.70	0.4854
Workgroup3	0.5471	0.3481	1.57	0.1167
Workgroup4	0.3945	0.3778	1.04	0.2969
Workgroup5	0.4139	0.3934	1.05	0.2933
Workgroup6	0.7918	0.3931	2.01	0.0445
Workgroup7	1.5559	1.1345	1.37	0.1709
Introversion	0.4555	0.1598	2.85	0.0046
Preferred time	-0.2985	0.1214	-2.46	0.0143
Works_noise	0.0066	0.1913	0.03	0.9723
Work_collagues2	0.4132	0.2794	1.48	0.1398
Work_collagues3	0.9941	0.3332	2.98	0.0030
Days_concept	0.1228	0.1221	1.01	0.3149

Further exploration of the covariates revealed that ratings of engagement increased by.4% for each year of participant age (*est*. = 0.048, *se* = 0.019 *p* = 0.011). Participants in the finance department reported 8% higher average engagement (*est*. = 0.791814 *se* = 0.393079 *p* = 0.045). Participants who identified themselves as extroverts reported 5% higher engagement than those who identified as introverts or halfway between (*est*. = 0.455, *se* = 0.160, *p* = 0.014). Participants who identified themselves as morning people reported 3% higher engagement than those who identified as afternoon or evening people (*est*. = -0.298, *se* = 0.121, *p* = 0.014). Participants whose tasks required high levels of collaboration reported 9% higher engagement than those whose tasks required medium or low collaboration (*est*. = 0.994, *se* = 0.333, *p* = 0.003).

#### Enjoyment

A linear model revealed a significant effect of office design on participant enjoyment of the workspace (*F* = 15.742, *p* < 0.001) (Table 9). Participants reported 11% more enjoyment in the Zoned open-plan design than in the Open-plan design (*est*. = 1.071, *se* = 0.364, *p* = 0.003). Conversely, participants reported 9% lower enjoyment in the Activity based design than in the Open-plan design (*est*. = -0.896, *se* = 0.378, *p* = 0.018). The Team office design did not receive significantly different enjoyment responses to the Open-plan design (*est*. = 0.649, *se* = 0.355, *p* = 0.068) (Table 10).

**Table 9:** ANOVA table for enjoyment.

	Df	Deviance	Resid. Df	Resid. Dev	F	Pr(>F)
NULL			507	3974.46		
area	3	313.85	504	3660.61	15.74	0.0000
group	3	22.55	501	3638.06	1.13	0.3360
previous	4	95.28	497	3542.78	3.58	0.0068
Gender	1	30.10	496	3512.68	4.53	0.0338
Age	1	20.63	495	3492.05	3.10	0.0787
Nationality	10	147.65	485	3344.40	2.22	0.0157
Workgroup	6	91.84	479	3252.56	2.30	0.0334
Introversion	1	22.16	478	3230.41	3.33	0.0685
Preferred time	1	34.38	477	3196.03	5.17	0.0234
Works_noise	1	0.02	476	3196.01	0.00	0.9562
Work_collagues	2	5.49	474	3190.51	0.41	0.6617
Days_concept	1	47.16	473	3143.36	7.10	0.0080

**Table 10:** GLM results for enjoyment.

	Estimate	Std. Error	t value	Pr(>|t|)
(Intercept)	3.3016	1.3294	2.48	0.0134
areaZONE	1.0706	0.3637	2.94	0.0034
areaACTIVE	-0.8962	0.3779	-2.37	0.0181
areaTEAM	0.6490	0.3552	1.83	0.0683
group2	0.2798	0.4751	0.59	0.5561
group3	-0.3042	0.4119	-0.74	0.4606
group4	-0.0913	0.4342	-0.21	0.8335
previousControl	0.6943	0.4258	1.63	0.1037
previousACTIVE	0.2125	0.4541	0.47	0.6401
previousTEAM	-0.0559	0.4634	-0.12	0.9040
previousNone	0.9954	0.3882	2.56	0.0107
Gender2	0.3051	0.2695	1.13	0.2580
Age	0.0340	0.0226	1.50	0.1337
Nationality2	-0.4447	0.8085	-0.55	0.5826
Nationality3	1.1329	0.9132	1.24	0.2154
Nationality4	0.1324	0.7193	0.18	0.8541
Nationality5	-0.3345	0.7770	-0.43	0.6670
Nationality6	0.3196	1.1099	0.29	0.7735
Nationality7	-0.5964	1.5104	-0.39	0.6931
Nationality8	-0.7918	0.8184	-0.97	0.3338
Nationality9	0.7759	0.7969	0.97	0.3308
Nationality10	-1.2784	1.0779	-1.19	0.2362
Nationality11	-0.7409	0.8941	-0.83	0.4077
Workgroup2	0.0579	0.6111	0.09	0.9246
Workgroup3	0.4162	0.4229	0.98	0.3255
Workgroup4	0.9681	0.4591	2.11	0.0355
Workgroup5	0.2281	0.4780	0.48	0.6335
Workgroup6	0.3640	0.4776	0.76	0.4464
Workgroup7	2.2846	1.3784	1.66	0.0981
Introversion	0.2531	0.1942	1.30	0.1932
Preferred time	-0.3629	0.1475	-2.46	0.0143
Works_noise	0.0123	0.2325	0.05	0.9580
Work_collagues2	-0.1712	0.3394	-0.50	0.6143
Work_collagues3	0.0910	0.4049	0.22	0.8222
Days_concept	0.3951	0.1483	2.66	0.0080

Of the covariates, the effect of the previous wave (*F* = 3.584, *p* < 0.001), gender (*F* = 15.742, *p* = 0.007), nationality (*F* = 2.222, *p* = 0.016), workgroup (*F* = 2.3033, *p* = 0.033), preferred time of day to work (*F* = 5.173, *p* = 0.023), and days spent in the concept (*F* = 7.096, *p* = 0.008) were all significant in the model.

Further exploration of the covariates revealed that the carryover effect of the previous office design was only significant in the first wave (i.e. when there was no previous design), such that participants in their first wave of the experiment reported 10% higher enjoyment of the workspace, regardless of office design (*est*. = 0.995, *se* = 0.388, *p* = 0.010). Participants who identified as female reported 3% higher enjoyment than participants who identified as male (*est*. = 0.305, *se* = 0.269, textitp = 0.258). Participants who worked in the HR department reported 10% higher enjoyment than those who worked in other departments (*est*. = 0.968, *se* = 0.459, *p* = 0.035). Participants who identified themselves as morning people reported 4% higher enjoyment of the workspace than those who identified as afternoon or evening people (*est*. = -0.363, *se* = 0.148, *p* = 0.0143). Each additional day participants had spent in the concept was associated with 4% higher enjoyment (*est*. = 0.395, *se* = 0.148, *p* = 0.008).

#### Environment

A series of linear models revealed no significant effects of office design on aggregated environmental variables (*F* = 20.629, df = 3) (Table 11). However, the Open-plan design recorded sound peaks outside of healthy ranges 20% more often than the Team Office design (*est*. = 0.195, *se* = 0.029, *p* = < 0.001), 29% more often than the Zoned open-plan design (*est*. = 0.291, *se* = 0.030, *p* = < 0.001), and 27% more often than the Activity based office design (*est*. = 0.269, *se* = 0.030, *p* = < 0.001) (Tables 12 and 13).

**Table 11:** ANOVA table for environment.

	Df	Deviance	Resid. Df	Resid. Dev	F	Pr(>F)
NULL			63	4.20		
area	3	0.12	60	4.08	0.55	0.6511
wave	1	0.05	59	4.03	0.74	0.3949
previous	4	0.07	55	3.96	0.23	0.9178

**Table 12:** ANOVA results for noise.

	Df	Deviance	Resid. Df	Resid. Dev	Pr(>Chi)
NULL			15	0.83	
area	3	0.76	12	0.07	0.0000
wave	1	0.00	11	0.07	0.7044
previous	4	0.03	7	0.04	0.3616

**Table 13:** GLM results for noise.

	Estimate	Std. Error	t value	Pr(>|t|)
(Intercept)	0.2514	0.1005	2.50	0.0409
areaZONE	0.5812	0.0580	10.02	0.0000
areaACTIVE	0.5089	0.0580	8.78	0.0001
areaTEAM	0.3492	0.0580	6.02	0.0005
wave	-0.0183	0.0278	-0.66	0.5318
previous Control	-0.0997	0.0669	-1.49	0.1801
previous ACTIVE	-0.0892	0.0669	-1.33	0.2245
previous TEAM	-0.0940	0.0669	-1.40	0.2031
previous None	-0.1538	0.0826	-1.86	0.1048

The Activity based design recorded an average temperature that was 0.4% lower than the Open-plan design, which was statistically significant (*est*. = -0.041, *se* = 0.014, *p* = 0.025) (Table 14). No significant differences were found for light, humidity, or air quality between the office space designs.

**Table 14:** GLM results for environment.

	Estimate	Std. Error	t value	Pr(>|t|)
(Intercept)	0.6843	0.1718	3.98	0.0002
areaZONE	0.1275	0.0991	1.29	0.2034
areaACTIVE	0.0879	0.0991	0.89	0.3787
areaTEAM	0.0846	0.0991	0.85	0.3969
wave	0.0060	0.0474	0.13	0.9005
previous Control	-0.0463	0.1144	-0.40	0.6872
previous ACTIVE	-0.0387	0.1144	-0.34	0.7362
previous TEAM	-0.0354	0.1144	-0.31	0.7580
previous None	0.0755	0.1411	0.54	0.5948

#### Energy

Office design did not have a significant effect on self reported energy levels at work (*F* = 1.439, *p* = 0.231) (Tables 15 and 16).

**Table 15:** ANOVA table for energy.

	Df	Deviance	Resid. Df	Resid. Dev	F	Pr(>F)
NULL			507	3455.27		
area	3	25.53	504	3429.74	1.44	0.2306
group	3	33.72	501	3396.01	1.90	0.1286
previous	4	52.70	497	3343.31	2.23	0.0651
Gender	1	1.11	496	3342.20	0.19	0.6647
Age	1	10.59	495	3331.61	1.79	0.1816
Nationality	10	195.73	485	3135.88	3.31	0.0004
Workgroup	6	133.24	479	3002.64	3.75	0.0012
Introversion	1	110.19	478	2892.45	18.63	0.0000
Preferred time	1	29.31	477	2863.14	4.96	0.0265
Works_noise	1	4.16	476	2858.98	0.70	0.4022
Work_collagues	2	44.15	474	2814.83	3.73	0.0246
Days_concept	1	17.41	473	2797.41	2.94	0.0868

**Table 16:** GLM results for energy.

	Estimate	Std. Error	t value	Pr(>|t|)
(Intercept)	5.0804	1.2541	4.05	0.0001
areaZONE	0.5292	0.3431	1.54	0.1237
areaACTIVE	-0.2779	0.3565	-0.78	0.4360
areaTEAM	0.2222	0.3351	0.66	0.5077
group2	0.5850	0.4482	1.31	0.1925
group3	0.2945	0.3886	0.76	0.4489
group4	0.4194	0.4096	1.02	0.3065
previousControl	-0.4572	0.4017	-1.14	0.2556
previousACTIVE	-0.9805	0.4284	-2.29	0.0225
previousTEAM	-0.3483	0.4372	-0.80	0.4260
previousNone	-0.1866	0.3662	-0.51	0.6107
Gender2	0.0742	0.2542	0.29	0.7706
Age	0.0296	0.0213	1.39	0.1657
Nationality2	-0.5653	0.7627	-0.74	0.4589
Nationality3	-0.4366	0.8615	-0.51	0.6126
Nationality4	-1.5346	0.6786	-2.26	0.0242
Nationality5	-1.4810	0.7330	-2.02	0.0439
Nationality6	-1.1453	1.0471	-1.09	0.2746
Nationality7	-0.8873	1.4249	-0.62	0.5338
Nationality8	-2.4336	0.7721	-3.15	0.0017
Nationality9	-1.0266	0.7518	-1.37	0.1727
Nationality10	-3.2112	1.0168	-3.16	0.0017
Nationality11	-1.4520	0.8434	-1.72	0.0858
Workgroup2	-1.1439	0.5765	-1.98	0.0478
Workgroup3	-0.0247	0.3990	-0.06	0.9507
Workgroup4	-0.4181	0.4331	-0.97	0.3348
Workgroup5	1.3015	0.4510	2.89	0.0041
Workgroup6	0.3233	0.4506	0.72	0.4734
Workgroup7	-1.7704	1.3004	-1.36	0.1740
Introversion	0.5184	0.1832	2.83	0.0049
Preferred time	-0.3156	0.1392	-2.27	0.0238
Works_noise	0.0600	0.2193	0.27	0.7846
Work_collagues2	0.3343	0.3202	1.04	0.2971
Work_collagues3	0.9811	0.3820	2.57	0.0105
Days_concept	0.2401	0.1399	1.72	0.0868

#### Flow

A linear model revealed a significant effect of office design on flow (*F* = 20.529, *p* < 0.001) (Table 17). Participants reported 12% higher flow in the Team office design (*est*. = 1.247, *se* = 0.281, *p* < 0.001) and 15% higher flow in the Zoned open-plan design (*est*. = 1.531, *se* = 0.288, *p* < 0.001) than in the Open-plan design. The Activity based office design did not receive significantly different flow responses to the Open-plan design (*est*. = -0.081, *se* = 0.299, *p* = 0.785).

**Table 17:** ANOVA table for flow.

	Df	Deviance	Resid. Df	Resid. Dev	F	Pr(>F)
NULL			507	2477.36		
area	3	256.06	504	2221.30	20.53	0.0000
group	3	42.67	501	2178.63	3.42	0.0172
previous	4	80.21	497	2098.42	4.82	0.0008
Gender	1	0.18	496	2098.23	0.04	0.8337
Age	1	0.15	495	2098.08	0.04	0.8470
Nationality	10	54.27	485	2043.81	1.31	0.2246
Workgroup	6	43.43	479	2000.39	1.74	0.1097
Introversion	1	4.43	478	1995.95	1.07	0.3024
Preferred time	1	4.37	477	1991.58	1.05	0.3057
Works_noise	1	0.00	476	1991.58	0.00	0.9900
Work_collagues	2	0.53	474	1991.06	0.06	0.9386
Days_concept	1	24.41	473	1966.65	5.87	0.0158

Of the covariates, the effect of the previous wave (*F* = 4.823, *p* < 0.001) and the number of days spent in the concept (*F* = 5.871, *p* = 0.016) contributed significantly to the explanatory power of the model (Table 18).

**Table 18:** GLM results for flow.

	Estimate	Std. Error	t value	Pr(>|t|)
(Intercept)	4.1468	1.0515	3.94	0.0001
areaZONE	1.5306	0.2877	5.32	0.0000
areaACTIVE	-0.0815	0.2989	-0.27	0.7853
areaTEAM	1.2471	0.2810	4.44	0.0000
group2	0.5841	0.3758	1.55	0.1208
group3	0.2503	0.3258	0.77	0.4428
group4	0.0284	0.3435	0.08	0.9342
previousControl	0.0228	0.3368	0.07	0.9460
previousACTIVE	-0.2686	0.3592	-0.75	0.4550
previousTEAM	-0.4620	0.3666	-1.26	0.2082
previousNone	0.5592	0.3071	1.82	0.0692
Gender2	0.0300	0.2131	0.14	0.8881
Age	-0.0012	0.0179	-0.07	0.9454
Nationality2	0.0179	0.6395	0.03	0.9777
Nationality3	0.0198	0.7224	0.03	0.9782
Nationality4	-0.0993	0.5690	-0.17	0.8615
Nationality5	-0.5126	0.6146	-0.83	0.4047
Nationality6	0.4313	0.8779	0.49	0.6235
Nationality7	0.4380	1.1947	0.37	0.7141
Nationality8	-0.4975	0.6474	-0.77	0.4426
Nationality9	0.6371	0.6304	1.01	0.3127
Nationality10	-0.5490	0.8526	-0.64	0.5200
Nationality11	-0.4276	0.7072	-0.60	0.5457
Workgroup2	0.7437	0.4833	1.54	0.1246
Workgroup3	0.4084	0.3345	1.22	0.2227
Workgroup4	-0.0763	0.3631	-0.21	0.8337
Workgroup5	0.3967	0.3781	1.05	0.2947
Workgroup6	0.3383	0.3778	0.90	0.3709
Workgroup7	2.0534	1.0903	1.88	0.0603
Introversion	0.1402	0.1536	0.91	0.3618
Preferred time	-0.1406	0.1167	-1.20	0.2290
Works_noise	0.0220	0.1839	0.12	0.9050
Work_collagues2	-0.0745	0.2685	-0.28	0.7816
Work_collagues3	-0.0951	0.3203	-0.30	0.7666
Days_concept	0.2843	0.1173	2.42	0.0158

Further exploration of the covariates revealed that the carryover effect of the previous office design was only significant in the first wave (i.e. when there was no previous design), such that participants in their first wave of the experiment reported 6% higher flow, regardless of office design (*est*. = 0.559, *se* = 0.307, *p* = 0.069). Each additional day participants had spent in the concept was associated with 3% higher flow (*est*. = 0.284, *se* = 0.117, *p* = 0.016).

#### Productivity

A linear model revealed a significant effect of office design on productivity (*F* = 31.570, *p* < 0.001) (Table 19). Participants reported reported 10% higher productivity in the Team office (*est*. = 1.032, *se* = 0.394, *p* = 0.009) and 17% higher productivity in the Zoned open-plan (*est*. = 1.715, *se* = 0.404, *p* < 0.001) designs than in the Open-plan design. Conversely, participants reported 14% lower productivity in the Activity based design than in the Open-plan design (*est*. = -1.424, *se* = 0.420, *p* < 0.001).

**Table 19:** ANOVA table for productivity.

	Df	Deviance	Resid. Df	Resid. Dev	F	Pr(>F)
NULL			507	5159.63		
area	3	776.76	504	4382.88	31.57	0.0000
group	3	89.46	501	4293.42	3.64	0.0129
previous	4	118.69	497	4174.73	3.62	0.0064
Gender	1	10.53	496	4164.20	1.28	0.2577
Age	1	4.01	495	4160.19	0.49	0.4848
Nationality	10	113.04	485	4047.15	1.38	0.1871
Workgroup	6	82.58	479	3964.58	1.68	0.1244
Introversion	1	15.61	478	3948.97	1.90	0.1684
Preferred time	1	27.91	477	3921.06	3.40	0.0657
Works_noise	1	1.71	476	3919.35	0.21	0.6483
Work_collagues	2	3.40	474	3915.95	0.21	0.8127
Days_concept	1	36.69	473	3879.26	4.47	0.0350

Of the covariates, the effect of having no previous design (*F* = 3.618, *p* = 0.006), and the number of days spent in the concept (*F* = 4.473, *p* = 0.035) had a significant effect on the explanatory power of the model (Table 20). Further exploration of the covariates revealed that participants reported 9.4% higher productivity in their first wave of the experiment regardless of office design (*est*. = 0.943, *se* = 0.431, *p* = 0.029). Each additional day participants had spent in the concept was associated with 3.5% higher productivity (*est*. = 0.349, *se* = 0.165, *p* = 0.035).

**Table 20:** GLM results for productivity.

	Estimate	Std. Error	t value	Pr(>|t|)
(Intercept)	2.9022	1.4768	1.97	0.0500
areaZONE	1.7155	0.4040	4.25	0.0000
areaACTIVE	-1.4244	0.4198	-3.39	0.0007
areaTEAM	1.0320	0.3946	2.62	0.0092
group2	0.9864	0.5278	1.87	0.0622
group3	0.3250	0.4576	0.71	0.4779
group4	0.1484	0.4824	0.31	0.7585
previousControl	0.6634	0.4730	1.40	0.1614
previousACTIVE	0.6431	0.5045	1.27	0.2030
previousTEAM	-0.3842	0.5148	-0.75	0.4559
previousNone	0.9427	0.4313	2.19	0.0293
Gender2	0.1976	0.2993	0.66	0.5095
Age	0.0243	0.0251	0.97	0.3340
Nationality2	0.0226	0.8981	0.03	0.9799
Nationality3	0.1055	1.0145	0.10	0.9173
Nationality4	0.1080	0.7991	0.14	0.8925
Nationality5	-0.2793	0.8631	-0.32	0.7464
Nationality6	0.9474	1.2330	0.77	0.4426
Nationality7	1.2873	1.6779	0.77	0.4433
Nationality8	-0.7263	0.9092	-0.80	0.4248
Nationality9	0.7924	0.8853	0.90	0.3712
Nationality10	-0.8178	1.1974	-0.68	0.4950
Nationality11	-0.5067	0.9932	-0.51	0.6102
Workgroup2	0.8589	0.6788	1.27	0.2064
Workgroup3	0.6730	0.4698	1.43	0.1527
Workgroup4	0.6541	0.5100	1.28	0.2003
Workgroup5	0.5995	0.5310	1.13	0.2595
Workgroup6	0.6745	0.5306	1.27	0.2043
Workgroup7	0.1987	1.5313	0.13	0.8968
Introversion	0.1671	0.2157	0.77	0.4391
Preferred time	-0.3223	0.1639	-1.97	0.0498
Works_noise	0.0839	0.2583	0.32	0.7455
Work_collagues2	0.1622	0.3771	0.43	0.6674
Work_collagues3	0.3092	0.4498	0.69	0.4921
Days_concept	0.3485	0.1648	2.11	0.0350

#### Commits

Office design was not found to have an effect on the number of git commits made by participants (*χ*^2^ = 0.372, df = 259) (Tables 21 and 22).

**Table 21:** ANOVA table for git commits.

	Df	Deviance	Resid. Df	Resid. Dev	Pr(>Chi)
NULL			262	2311.10	
area	3	3.13	259	2307.97	0.3724
wave	3	196.91	256	2111.06	0.0000
group	3	200.57	253	1910.49	0.0000
previous	3	10.97	250	1899.52	0.0119

**Table 22:** GLM results for git commits.

	Estimate	Std. Error	z value	Pr(>|z|)
(Intercept)	1.8550	0.0753	24.63	0.0000
area ZONE	0.1343	0.0643	2.09	0.0368
area ACTIVE	0.0099	0.0699	0.14	0.8879
area TEAM	-0.0464	0.0629	-0.74	0.4611
wave 2	0.0529	0.0759	0.70	0.4855
wave 3	0.5135	0.0758	6.77	0.0000
wave 4	-0.2415	0.0824	-2.93	0.0034
group B	0.5327	0.0707	7.53	0.0000
group C	0.0496	0.0640	0.78	0.4378
group D	0.6835	0.0661	10.33	0.0000
previous Control	0.0370	0.0776	0.48	0.6336
previous ACTIVE	0.0382	0.0704	0.54	0.5877
previous TEAM	-0.1834	0.0834	-2.20	0.0279

#### Occupancy

A linear model revealed a significant effect of office design on hourly occupancy (*F* = 20.842, *p* = < 0.001) (Table 23). Sensors in the Activity based design recorded a 5% higher hourly occupancy than the Open-plan design (*est*. = 0.052, *se* = 0.008, *p* = < 0.001) (Table 24). There were no significant differences in hourly occupancy between the Open-plan design and the Zoned open-plan or Team office designs.

**Table 23:** ANOVA table for occupancy.

	Df	Deviance	Resid. Df	Resid. Dev	F	Pr(>F)
NULL			1261	11.89		
area	3	0.54	1258	11.35	20.63	0.0000
previous	4	0.24	1254	11.11	6.91	0.0000
wave	1	0.20	1253	10.91	23.41	0.0000
group	3	0.04	1250	10.87	1.53	0.2043

**Table 24:** GLM results for occupancy.

	Estimate	Std. Error	t value	Pr(>|t|)
(Intercept)	0.3168	0.0142	22.24	0.0000
areaZONE	0.0152	0.0080	1.89	0.0590
areaACTIVE	0.0519	0.0076	6.79	0.0000
areaTEAM	0.0100	0.0077	1.30	0.1953
previousControl	0.0356	0.0091	3.90	0.0001
previousACTIVE	0.0196	0.0095	2.06	0.0396
previousTEAM	0.0131	0.0091	1.45	0.1475
previousNone	-0.0001	0.0114	-0.01	0.9917
wave	-0.0184	0.0037	-4.93	0.0000
group 2	0.0003	0.0077	0.03	0.9734
group 3	-0.0150	0.0079	-1.89	0.0586
group 4	-0.0041	0.0077	-0.54	0.5898

Of the covariates, both the wave of the study (*F* = 18.871, *p* = < 0.001) and the previous design (*F* = 6.981, *p* = < 0.001) had a significant effect on hourly occupancy.

Further exploration of the covariates revealed that the carryover effect of the previous office design was significant in the first wave, such that sensors recorded 6% higher hourly occupancy in the first wave of the experiment, across all designs (*est*. = 0.062, *se* = 0.009, *p* = < 0.001). Additionally, moving out of the Open-plan design was associated with a 4% increase in hourly occupancy (*est*. = 0.036, *se* = 0.009, *p* = < 0.001). Sensors recorded 2% lower hourly occupancy for each wave of the experiment (*est*. = -0.018, *se* = 0.004, *p* = < 0.001), indicating that hourly occupancy overall decreased as the experiment progressed.

## Discussion

While this research yielded many results, three specific findings should be of interest to researchers and practitioners.

First, our results demonstrated that office designs can affect employees’ satisfaction, engagement, enjoyment, flow, and productivity. The Zoned open-plan design was highest rated in terms of employees’ satisfaction, enjoyment, flow, and self-reported productivity. The Team Office design was highest rated for engagement and additionally was higher rated for satisfaction, flow, and self-reported productivity than the Open-plan or Activity based designs. The Open-plan office did not perform better than any other office designs, on any of these outcomes. Additionally, moving out of the Open-plan office was associated with an increase in desk occupancy, suggesting that employees’ preference for other designs was matched by their actual behaviour in showing up more when they no longer had to participate in the Open-plan design of the experiment. Finally, the sensors in the Activity based design reported a 5% increase in the proportion of desks that were occupied compared to other designs. However, given that the Activity based design included 27% fewer workstations than other designs, we interpret this finding to mean that desks were harder to find in this design, rather than that more people were occupying the area. The Activity based design did not perform better than other designs on any other outcomes and was worse than the Open-plan office for employee satisfaction, enjoyment, and productivity.

Second, noise was the main environmental variable to differentiate office designs. The noise levels in the Open-plan office were within healthy ranges 20% and 30% less often than in the other three designs, indicating a large difference between Open-plan and other designs in terms of noise. Thus, part of the reason for employees’ more positive ratings of other office designs may be that these are less distracting, and more comfortable, compared to Open-plan. While it is well established that quieter work spaces facilitate productivity, this research demonstrates the critical role of office design in managing noise.

Third, the consistency of results demonstrated that, in general, office design can be experimentally tested in a working office without disrupting business continuity. The cross-over design method proved to be a practical and informative method for office experimentation. However, we did observe higher enjoyment, flow, and occupancy when participants had not been in any other experimental design previously. This observation suggests that simply running an experiment on office design with employees may have a positive effect on enjoyment of the space, engagement, and occupancy. While this experimental design is noted to be be not as efficient as other designs [117], the advantages in overcoming commercial and logistic challenges make it a valuable tool in the practitioners toolkit.

We additionally observed some interesting findings from our exploratory analysis of the covariates. First, employees differed in their reported engagement with their work. Specifically, employees whose jobs required high levels of collaboration also reported high levels of engagement with their work, and this effect was slightly larger than the effect for office design. Additionally, employees identifying as extroverts and employees identifying as morning people reported higher engagement with their work. Finally, some employees reported higher enjoyment of their work than others, specifically, women, people working in HR, and people whose work required high levels of collaboration. However, in general, the effect of office design was larger than that of any employee covariates, suggesting that office design is of equal or greater importance to employee satisfaction, engagement, flow, and productivity than any personal variables.

We encountered some challenges in the process of conducting this experiment. First, the real-world setting prevented fully randomized selection of participants, as certain teams were unable or unwilling to participate, and teams could not be separated for the experiment and so allocation was randomized at the team level. We controlled for this experimentally through a randomized exposure pattern, and statistically by explicitly including a term for group effects in the statistical models. Second, while the study included a diverse group in terms of nationality, the sample was on average younger than the general population, as they were drawn from a specific corporate population. Results may therefore not generalise to companies with an older population. We recommend repeating the experiment in other companies with different age ranges. Third, the exposure of participants to designs could not be strictly controlled. For example, if a meeting room could not be booked within one experimental area, sometimes a team might book a meeting room in a different experimental area. This is unavoidable in a real world setting, but did not happen commonly, and participants were discouraged from the practice. Fourth, as a corporate research project, anonymity was especially important and this prevented us from tracking individual respondents from one design to the next. Finally, the survey was conducted in English, and while English is the language spoken at the office, this may have introduced some bias in a population that speaks many languages natively.

## Conclusion

This paper presents a cross-over experimental evaluation of open office designs in a working technology company. It builds on previous studies of employee experience and behaviour in the office by combining many different factors to determine an optimal office design. We found that Zoned open-plan and Team office designs improved employee satisfaction, enjoyment, flow, and productivity, while Activity based and Open-plan designs performed poorly by comparison. The Open-plan office design was rated more poorly by employees, had higher levels of unsafe noise, and once employees no longer had to be in the Open-plan office design of the experiment, they spent more time at their desks. Given that the Open-plan design is used in many major technology companies, these findings should be noted by the wider industry as it suggests that many companies could benefit from redesigning their offices. The results suggest that office design can have a significant impact on employee productivity, health, and wellbeing across a range of factors. This effect should be considered and studied by companies faced with the decision of how to design their office spaces, and factored into budgeting and design stages.

## Supporting information

S1 File (ZIP)
