# Correction: The work environment pilot: An experiment to determine the optimal office design for a technology company

**DOI:** 10.1371/journal.pone.0235428

**Published:** 2020-06-24

**Authors:** Jegar Pitchforth, Elizabeth C. Nelson, Marc van den Helder, Wouter Oosting

The second author's name is incorrect. The correct name is: Elizabeth C. Nelson. The correct citation is: Pitchforth J, Nelson EC, van den Helder M, Oosting W (2020) The work environment pilot: An experiment to determine the optimal office design for a technology company. PLoS ONE 15(5): e0232943. https://doi.org/10.1371/journal.pone.0232943

The ORCID iD is missing for the second author. Please see the author’s ORCID iD here:

- Author Elizabeth C. Nelson’s ORCID iD is: 0000-0001-7422-1015 (https://orcid.org/0000-0001-7422-1015).
